# Predicting outcomes in patients with pulmonary hypertension using right ventricular global longitudinal strain versus tricuspid annular plane systolic excursion (TAPSE) and fractional area change: a retrospective analysis

**DOI:** 10.1186/s44348-025-00059-0

**Published:** 2025-10-30

**Authors:** Noura Alturaif, Emily Lin, Anirudh Sundararaghavan, Valentina Mercurio, Tucker Wilkinson, Thomas Hilton, Onyedika Ilonze, Khadijah Breathett, Jane Kabwe, Joseph Phiri, Brian Graham, Joan F. Hilton, Andrew Mihalek, Nicholas Ashur, Daniel Patterson, Kenneth Bilchick, Sula Mazimba

**Affiliations:** 1 Lung Health Department, Organ Transplant Centre, Faisal Specialist Hospital and Research Centre, Riyadh, King Saudi Arabia; 2 University of Virginia School of Medicine, Charlottesville, VA USA; 3 Department of Internal Medicine, University of Virginia, Charlottesville, VA USA; 4 Department of Translational Medical Sciences, University of Naples Federico II, Naples, Italy; 5 Cardiovascular Department, Indiana University School of Medicine, Indiana, IN USA; 6 Department of Anesthesia and Critical Care, National Heart Hospital, Lusaka, Zambia; 7 Department of Adult Cardiology, National Heart Hospital, Lusaka, Zambia; 8 Pulmonary and Critical Care Medicine, University of California San Francisco, San Francisco, CA USA; 9 Department of Epidemiology and Biostatistics, University of California San Francisco, San Francisco, CA USA; 10 Department of Pulmonary and Critical Care Medicine, University of Virginia, Charlottesville, VA USA; 11 Cardiovascular Department, University of Virginia, Charlottesville, VA USA; 12 Morningstar Clinic, Lusaka, Zambia; 13 AdventHealth Transplant Institute, Orlando, FL USA

**Keywords:** Pulmonary hypertension, Echocardiography, Strain, Right ventricular function, Outcome

## Abstract

**Background:**

Pulmonary hypertension (PH) is a progressive clinical condition that eventually leads to right ventricular (RV) failure. RV function is the primary determinant of morbidity and mortality in patients with PH. RV global longitudinal strain (RVGLS) is a promising echocardiographic metric used to assess RV function in this setting. Our study aimed to compare the ability of RVGLS, tricuspid annular plane systolic excursion (TAPSE) and fractional area change (FAC) to predict adverse outcomes in patients with PH.

**Methods:**

We retrospectively evaluated 315 patients with PH of diverse etiologies with 62% constitute of WHO group 2 disease, who were followed at the PH clinic at the University of Virginia, from March 2012 to December 2018. We included all adult patients who met the hemodynamic definition of PH with right heart catheterization and who underwent echocardiography within 1 month of each other.

**Results:**

Approximately half of the cohort was female, with a mean age of 64 ± 14 years. We found a strong correlation between RVGLS and FAC (r =  − 0.55, P < 0.001). Furthermore, there was a significant correlation between RVGLS and invasive hemodynamics. Compared with the TAPSE, the RVGLS stratified by quartiles was associated with mortality at 5 years and hospitalization.

**Conclusion:**

RVGLS is an echocardiographic marker that correlates closely with FAC and invasive pulmonary hemodynamics. In this study, both RVGLS and FAC were associated with 5-year mortality, whereas TAPSE was not. Notably, only RVGLS showed a significant association with hospitalization, suggesting that it may provide additional prognostic value in patients with PH.

**Supplementary Information:**

The online version contains supplementary material available at 10.1186/s44348-025-00059-0.

## Background

Pulmonary hypertension (PH) is a condition characterized by an increase in mean pulmonary artery pressure (mPAP) > 20 mmHg measured during right heart catheterization (RHC) that progressively leads to right ventricular (RV) failure and ultimately death. The current evidence indicates that better outcomes result from early disease identification and timely intervention [1]. RV function is a key determinant of prognosis in patients with PH, in addition to its specific etiology, and echocardiography has remained the cornerstone for the noninvasive assessment of RV function [2, 3]. Various echocardiographic metrics have been used to assess RV function and pulmonary hemodynamics. These metrics include tricuspid annular plane systolic excursion (TAPSE), fractional area change (FAC), and speckle-tracking-derived RV global longitudinal strain (RVGLS). These parameters provide essential information for diagnosing, monitoring, and managing patients with PH. However, the RV has a complex geometrical structure that renders assessment of function very challenging. Although several conventional echocardiographic indices can indicate RV systolic function, no echocardiographic index represents intrinsic myocardial changes and early RV dysfunction. Strain echocardiography is a promising modality for measuring myocardial deformation [4]. There is growing evidence that it can detect subclinical myocardial changes in the early disease stages and may enhance risk stratification [5]. RV strain has been studied in patients with postcapillary PH, such as those with chronic left ventricular failure, and those with precapillary PH, whether classified as group 1 pulmonary arterial hypertension (PAH) by the World Health Organization (WHO) or PH associated with lung disease, and was able to predict future outcomes in those patients [6–8]. RVGLS is thus a promising tool for the assessment of RV function in patients with PH. RVGLS has the advantage that it is not dependent on geometrical assumptions and can measure regional myocardial deformation patterns. Recent studies suggest that, compared with other echocardiographic parameters, RVGLS may be superior in assessing RV function [9–13]. This study aimed to assess the ability of the RVGLS, TAPSE, and FAC to predict adverse outcomes in patients with PH.

## Methods

### Patient population

We retrospectively included 315 patients with PH who underwent longitudinal follow-up at the PH clinic at the University of Virginia, Charlottesville, VA, USA, between March 2012 and December 2018. Clinical data were obtained through retrospective review of participants’ electronic medical records from the time of enrollment through 2022, allowing for up to 5 years of follow-up.

We included all adult PH patients (≥ 18 years) with a mPAP > 20 mmHg who underwent RHC and echocardiography within 1 month. PH is defined by mPAP > 20 mmHg at rest. The updated hemodynamic definition of PH includes pulmonary artery wedge pressure (PAWP) and pulmonary vascular resistance (PVR) to classify patients into precapillary, postcapillary, or combined disease. Precapillary PH is defined by PVR > 2 Wood units (WU) and PAWP ≤ 15 mmHg. Postcapillary PH is defined by PVR ≤ 2 WU with elevated PAWP (> 15 mmHg). Combined PH is defined when both PVR and PAWP are elevated (PVR > 2 WU and PAWP > 15 mmHg, respectively) [14]. The REVEAL score, a risk stratification tool used to predict mortality in patients with PAH, was applied in this cohort to patients with WHO Group 1 and Group 4 pulmonary hypertension.

**Fig. 1:**
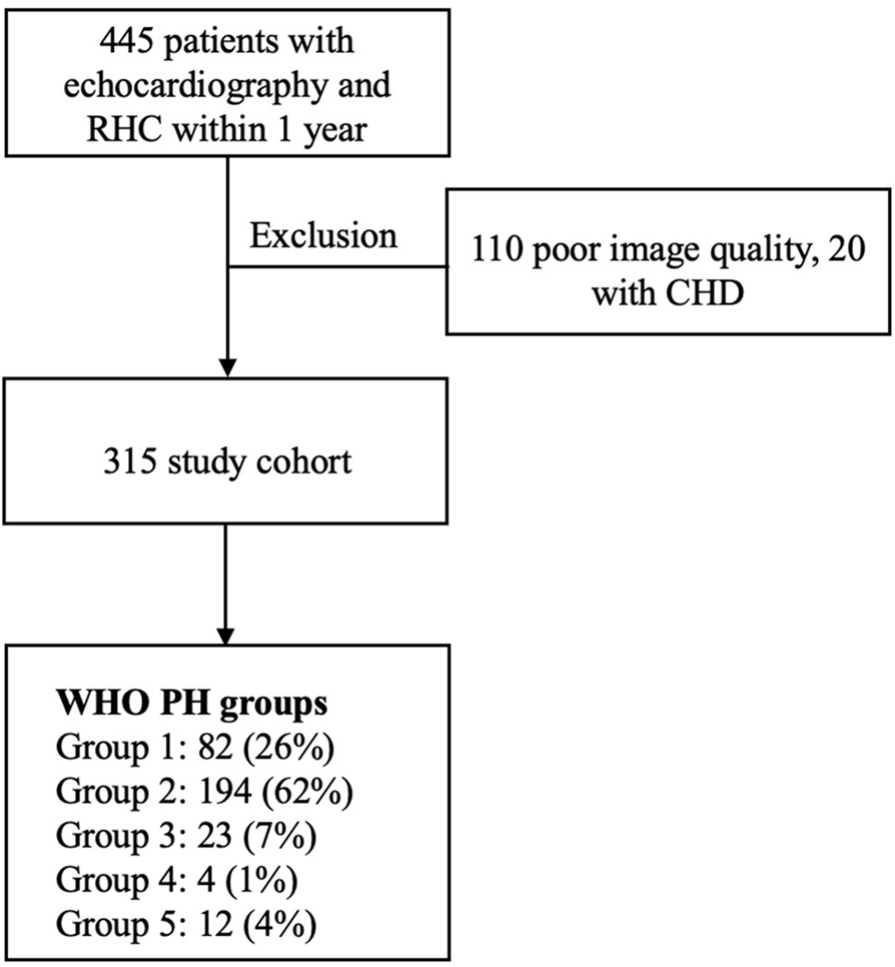
Study flow diagram. CHD, congenital heart disease; PH, pulmonary hypertension; RHC, right heart catheterization; WHO, World Health Organization

We excluded those with congenital heart disease–associated PAH, as well as those with poor echocardiographic image quality that precluded RVGLS and/or FAC measurement, as shown in Fig. 1.

### Echocardiography

Images were obtained while the patient was in the left lateral decubitus position. Experienced sonographers, using standard echocardiographic views, obtained transthoracic echocardiographic images. Left ventricular volume and function were evaluated per guidelines. RV dimensions and function were evaluated by measuring the TAPSE. The RV FAC was measured in every patient retrospectively. The RV pressure was estimated by calculating the maximum velocity of the Tricuspid Regurgitant Jet (TRJ) on the basis of the modified Bernoulli equation [15]. The right atrial (RA) pressure was estimated by measuring the diameter and collapsibility of the inferior vena cava. By adding the RV pressure to the RA pressure, the systolic PAP (sPAP) was estimated using the tricuspid regurgitation velocity. Speckle-tracking analysis of the RV was retrospectively performed offline from the apical four-chamber view and dedicated RV focused views. The endocardial border of the RV free wall and interventricular septum was manually traced at end-systole. The region of interest was subsequently manually adjusted to the thickness of the myocardium to ensure proper tracking [16]. All images were obtained using Philips iE33 (Philips Medical Systems), Epiq 7CV (Philips Medical Systems), or GE Vivid E9 Ultrasound (GE). All studies were analyzed using Enterprise Imaging (AGFA HealthCare).

### Right heart catheterization

The RHC procedure was performed in the RHC laboratory based on clinical indications for hemodynamic assessment. RA, RV, sPAP, diastolic PAP, and mPAP, as well as PAWP, were measured in all patients. Cardiac output (CO) assessment was performed using indirect Fick and thermodilution methods. PVR was calculated using mPAP, PAWP, and CO. RHC was performed according to standard guidelines [14]. Provocative maneuvers such as fluid challenge, stationary bike peddles, and vasodilation testing were completed at the discretion of cardiologists.

### Statistical analysis

Continuous data are presented as the means and standard deviations and as the number of cases and percentages for categorical variables (*P* < 0.05). Pearson correlation was used to assess the correlation between echocardiographic metrics, including RVGLS, and hemodynamic variables. The quartile cutoffs used for RVGLS were based on the distribution within our cohort. The rationale for using quartile-based stratification was to capture potential nonlinear associations with outcomes, which are common in strain-derived measurements. Additionally, we used Spearman correlation to assess the type of correlation between those variables and the chi-square test to assess whether there was an association between RVGLS quartiles and hospitalization and death at 1 and 5 years. Mortality was defined as all-cause death, and hospitalization was defined as all-cause hospitalization. Mortality was modeled as a binary endpoint at a fixed 5-year horizon using logistic regression. Hospitalizations were modeled as a continuous outcome using linear regression coefficients, as the number of hospitalizations was treated as a continuous variable. Univariate linear regression was subsequently used to assess which RVGLS quartile was associated with poor outcomes. Then, we employed a modular approach to assess RVGLS in context with different clinical domains (demographics, PH classification, hemodynamics, and function). Analysis of variance was used to assess the association between RVGLS and poor outcomes since the index echocardiography. In addition, we used logistic regression and receiver operating characteristic analysis to identify which RVGLS quartile and/or value was best at predicting mortality. Analyses were performed using commercially available R ver. 4.5.0 (R Foundation for Statistical Computing).

## Results

We enrolled a total of 315 patients with complete hemodynamics and adequate echocardiographic images. Approximately half of the cohort was female (*n* = 185, 58.7%,), with a mean age of 64 ± 14 years. Among the cohort, 194 (61.6%) were categorized as WHO group 2 PH, and 82 (26.0%) were categorized as WHO group 1. Patient comorbidities are shown in Table 1. Most of the patients had New York Heart Association (NYHA) functional classes II (*n* = 135, 42.9%) and III (*n* = 138, 43.8%). Furthermore, as expected, most of the patients (n = 224, 71.1%) were not receiving pulmonary vasodilator therapy since most had WHO group 2 disease. The mean number of days between RHC and echocardiography was 8. The mean RVGLS, FAC, TAPSE, and TAPSE/sPAP were − 6.54% ± 3.75%, 26.0% ± 7.2%, 17.0 ± 0.7 mm, and 0.3 ± 0.2 mm/mmHg, respectively (Table 1) [17]. During the follow-up period from the index echocardiography, 231 patients experienced at least one hospitalization, accounting for a total of 589 hospitalizations over 5 years. There were 71 deaths within the first year and 120 deaths by 5 years.

**Table 1:** Baseline characteristics (n = 315)

Characteristic	Value	
Age (yr)	64 ± 14	
Female sex	185 (58.7)	
White race	228 (72.4)	
Weight (kg)	87 ± 24	
Pulmonary hypertension		
WHO group 1	82 (26.0)	
WHO group 2	194 (61.6)	
WHO group 3	23 (7.3)	
WHO group 4	4 (1.3)	
WHO group 5	12 (3.8)	
Comorbidity		
Diabetes mellitus	112 (35.6)	
Hypertension	223 (70.8)	
Heart failure with reduced ejection fraction	92 (29.2)	
Atrial fibrillation	84 (26.7)	
Chronic obstructive pulmonary disease	72 (22.9)	
Renal disease	171 (54.3)	
Pulmonary embolism	28 (8.9)	
NYHA functional class		
I	13 (4.1)	
II	135 (42.9)	
III	138 (43.8)	
IV	23 (7.3)	
REVEAL score (n = 86)^a^		
High	43 (50)	
Intermediate	25 (29.1)	
Low	18 (20.9)	
6MWT (min)	209 ± 66	
BNP (pg/mL)	315 ± 102	
DLCO (%)	42 ± 16	
Echocardiography metric		
TAPSE (mm)		17.0 ± 0.7
FAC (%)		26.0 ± 7.2
RVGLS (%)		− 6.54 ± 3.75
sPAP (mmHg)		57 ± 20
TAPSE/sPAP (mm/mmHg)		0.3 ± 0.2
Hemodynamic		
Right atrial pressure (mmHg)		12.0 ± 6.6
sPAP (mmHg)		61.5 ± 18.5
mPAP (mmHg)		39 ± 11.43
PAWP (mmHg)		19 ± 8.6
Thermodilution cardiac output (L/min)		5.8 ± 2
Thermodilution cardiac index (L/min/m^2^)		3 ± 1
Stroke Volume (mL/beat)		78 ± 29
Pulmonary vascular resistance (WU)		5 ± 4
No. of days between RHC and echocardiography		8.0 ± 8.5
Pulmonary vasodilator therapy		
None		224 (71.1)
Monotherapy		65 (20.6)
Combination therapy		26 (8.3)

Values are presented as mean ± standard deviation or number (%)

WHO, World Health Organization; NYHA, New York Heart Association; REVEAL, Registry to Evaluate Early and Long-term Pulmonary Arterial Hypertension Disease Management; 6MWT, 6-min walk test; BNP, B-type natriuretic peptide; DLCO, diffusing capacity of the lungs for carbon monoxide; TAPSE, tricuspid annular plane systolic excursion; FAC, fractional area change; RVGLS, right ventricular global longitudinal strain; sPAP, systolic pulmonary artery pressure; mPAP, mean pulmonary artery pressure; PAWP, pulmonary artery wedge pressure; SV, Stroke volume; WU, Woods unit; RHC, right heart catheterization;

^a^REVEAL score is a risk stratification tool used to predict mortality in patients with PAH [17]. REVEAL score calculation was feasible in a total of 86 patients

**Fig. 2:**
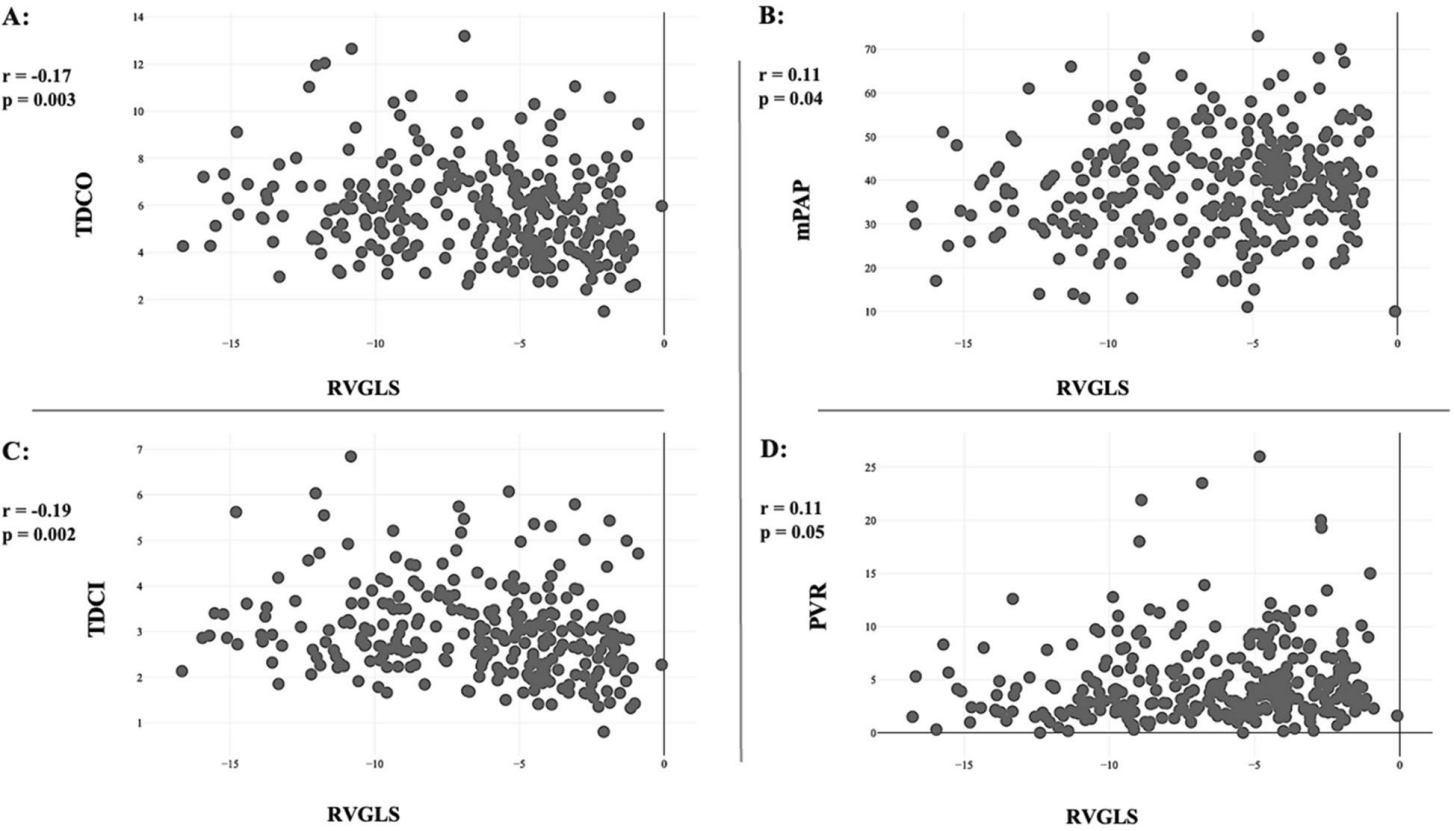
Correlation between right ventricular global longitudinal strain (RVGLS) and invasive hemodynamic parameters. (A) RVGLS versus thermodilution cardiac output (TDCO). (B) RVGLS versus mean pulmonary artery pressure (mPAP). (C) RVGLS versus thermodilution cardiac index (TDCI). (D) RVGLS versus pulmonary vascular resistance (PVR). Each panel displays the Pearson correlation coefficient (r) and the corresponding P-value

**Fig. 3:**
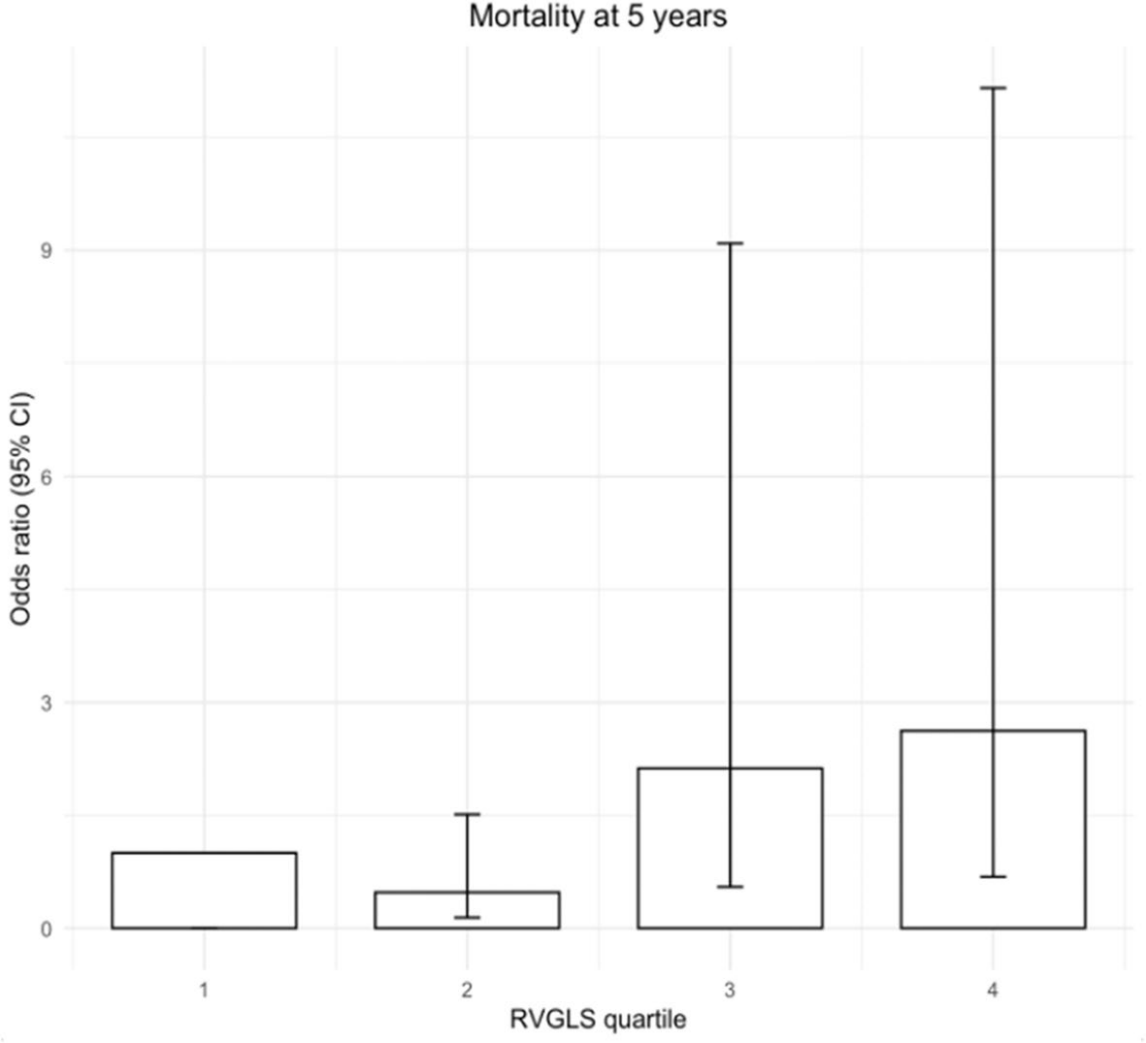
Association of right ventricular global longitudinal strain (RVGLS) quartiles with mortality at 5 years. Patient distribution across quartiles were as follows: quartile 1 (*n* = 79), quartile 2 (*n* = 77), quartile 3 (*n* = 79), and quartile 4 (*n* = 79)

RVGLS correlated strongly with FAC (r = –0.55, P < 0.001) but showed no correlation with TAPSE (r = –0.04, P = 0.76) or TAPSE/sPAP (r = –0.14, P = 0.22). In addition, there was a significant correlation between RVGLS and thermodilution CO, thermodilution cardiac index, mPAP, and PVR (Table 2, Fig. 2). In our detailed analysis of the relationship between RVGLS and CO, we specifically evaluated the association with stroke volume. Notably, a significant association between RVGLS and stroke volume emerged predominantly within the combined PH group (P < 0.001). Additionally, within this same group, a notable association was observed between RVGLS and the left ventricular ejection fraction, with P < 0.001 (Supplementary Table 1). Moreover, we found a strong association between RVGLS quartiles and 5-year mortality (odds ratio [OR] per quartile increase, 1.53; 95% confidence interval [CI], 1.03–2.23; P = 0.036), particularly in quartile 4 (RVGLS > –3.64) (Fig. 3).

**Table 2:** Correlations between RVGLS and other echocardiographic metrics and invasive hemodynamic

Correlation	Pearson correlation coefficient (r)	P-value
RVGLS and echocardiographic metrics		
FAC	− 0.55	< 0.001
TAPSE	− 0.04	0.76
TAPSE/sPAP	–0.14	0.22
RVGLS and hemodynamics		
mPAP	0.11	0.04
PVR	0.11	0.05
TDCO	− 0.17	0.003
TDCI	− 0.19	0.002
FAC and hemodynamics		
mPAP	− 0.24	< 0.001
PVR	–0.20	< 0.001
TDCD	0.25	< 0.001
TDCI	0.29	< 0.001
TAPSE and hemodynamics		
mPAP	–0.09	0.41
PVR	0.06	0.62
TDCD	0.42	< 0.001
TDCI	0.50	< 0.001

RVGLS, right ventricular global longitudinal strain; FAC, fractional area change; TAPSE, tricuspid annular plane systolic excursion; sPAP, systolic pulmonary artery pressure; mPAP, mean pulmonary artery pressure; PVR, pulmonary vascular resistance; TDCO, thermodilution cardiac output; TDCI, thermodilution cardiac index

**Fig. 4:**
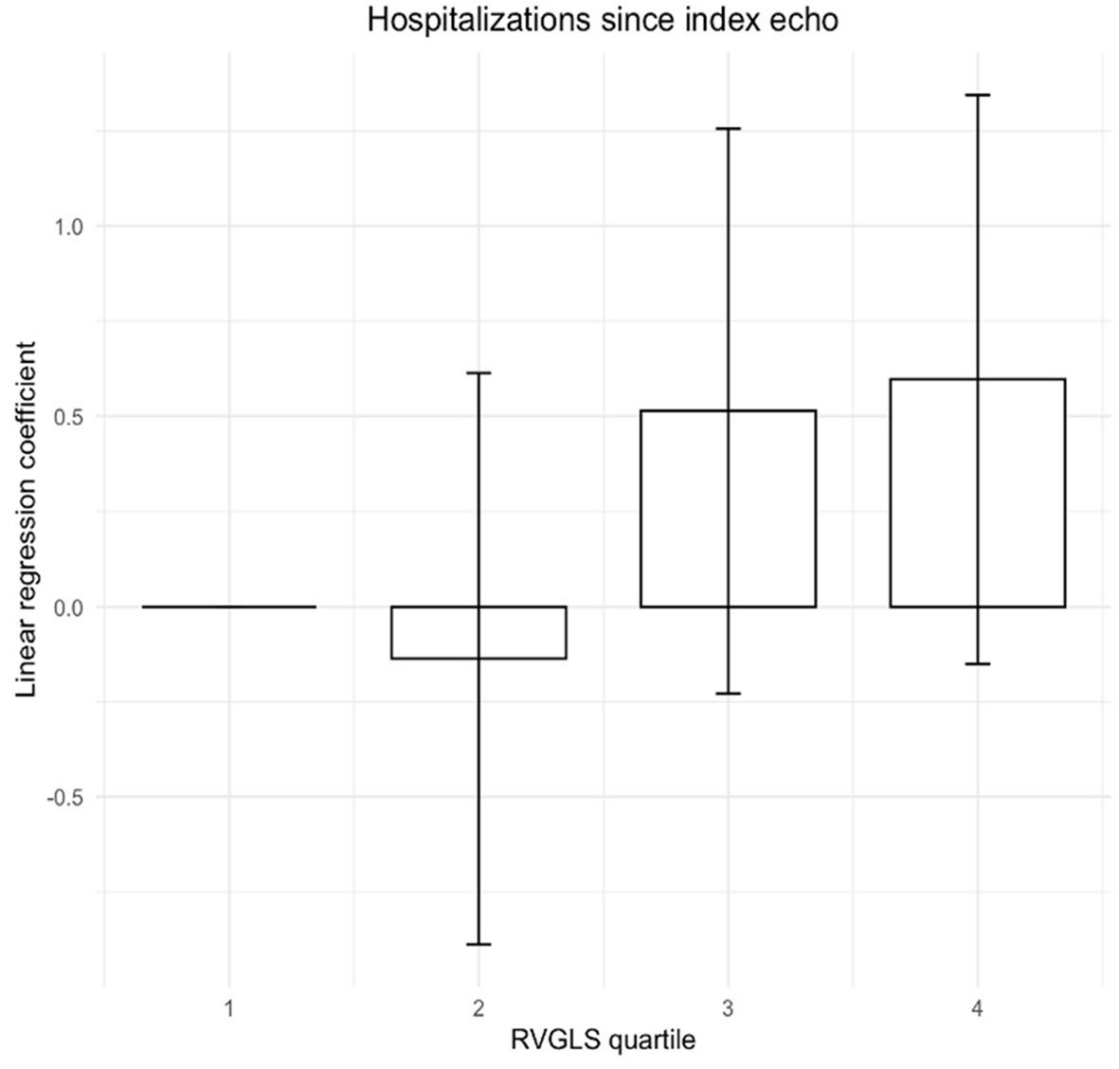
Association of right ventricular global longitudinal strain (RVGLS) quartiles with hospitalizations since the index echocardiography (echo). Patient distribution across quartiles were as follows: quartile 1 (*n* = 79), quartile 2 (*n* = 77), quartile 3 (n = 79), and quartile 4 (n = 79)

Using univariate linear regression, we found an association between RVGLS quartiles and hospitalization since the index echocardiography (intercept, 0.25; *P* = 0.041) (Fig. 4). However, there was no significant association between RVGLS and poor outcome (hospitalizations, death at 1 and 5 years) in each PH subtype, as shown in Table 3. Univariate analysis demonstrated that RVGLS was a significant predictor of adverse outcomes at 5 years (Table 4), with an OR of 1.53.

**Table 3:** RVGLS prediction of poor outcomes in each PH group

Variable	Coefficient	P-value
Precapillary PH (n = 111, 35.2%)		
Death at 1 yr	0.096	0.23
Death at 5 yr	0.062	0.53
Hospitalization	0.03	0.67
Postcapillary PH (n = 51, 16.2%)		
Death at 1 yr	0.12	0.26
Death at 5 yr	0.19	0.25
Hospitalization	0.01	0.90
Combined PH (n = 153, 48.6%)		
Death at 1 yr	0.019	0.70
Death at 5 yr	0.09	0.22
Hospitalization	0.08	0.14

RVGLS, right ventricular global longitudinal strain; PH, pulmonary hypertension

**Table 4:** Univariate analysis of independent predictors of all-cause mortality at 5 years

Predictor	All-cause mortality	
	OR (95% CI)	*P*-value
Age (yr)	1.04 (1.01–1.08)	0.03
Sex	1.09 (0.45–2.64)	0.11
NYHA functional class (III, IV)	5.76 (2.27–16.1)	< 0.001
Pulmonary vascular resistance	1.27 (1.07–1.58)	0.05
SVO_2_	0.91 (0.86–0.96)	< 0.001
Thermodilution cardiac index	0.59 (0.37–0.90)	0.003
Capillary type		
Precapillary PH	1	-
Postcapillary PH	0.83 (0.27–3.12)	0.76
Combined PH	1.09 (0.45–2.66)	0.85
RVGLS (%)	1.53 (1.03–2.30)	0.036
FAC (%)	0.91 (0.85–0.97)	0.008
TAPSE (mm)	2.79 (0.20–38.00)	0.44

OR, odds ratio; CI, confidence interval; NYHA, New York Heart Association; SVO_2_, venous oxygen saturation; PH, pulmonary hypertension; RVGLS, right ventricular global longitudinal strain; FAC, fractional area change; TAPSE, tricuspid annular plane systolic excursion

In the multivariable model (model 1), RVGLS remained independently associated with mortality (OR, 1.66; 95% CI, 1.10–2.57; P = 0.02), indicating a 66% increase in the odds of mortality per unit worsening in RVGLS. Importantly, RVGLS retained its prognostic value after adjusting for age, sex, and PH subtype (Table 5). Among clinical variables, NYHA functional class III–IV was strongly associated with mortality, conferring more than a fourfold increase in the odds of death (OR, 4.47).

**Table 5:** Multivariable analysis of independent predictors of all-cause mortality at 5 years

Predictor	All-cause mortality	
	OR (95% CI)	P-value
Model 1		
RVGLS (%)	1.66 (1.10–2.57)	0.02
Age (yr)	1.05 (1.01–1.09)	0.01
Sex	0.92 (0.36–2.34)	0.80
Model 2		
RVGLS (%)	1.54 (1.03–2.33)	0.04
Capillary type		
Precapillary PH	1	-
Postcapillary PH	1.11 (0.30–4.73)	0.87
Combined PH	1.04 (0.38–2.77)	0.94
Model 3		
RVGLS (%)	1.06 (0.93–1.21)	0.39
Pulmonary vascular resistance	1.19 (0.97–1.52)	0.14
SVO_2_	0.904 (0.83–0.97)	0.01
Thermodilution cardiac index	1.01 (0.56–1.84)	0.97
Model 4		
RVGLS (%)	1.38 (0.89–2.19)	0.15
NYHA functional class (III, IV)	4.47 (1.68–12.87)	0.004
LVEF (%)	1.11 (0.13–24.68)	0.90

RVGLS, right ventricular global longitudinal strain; PH, pulmonary hypertension; SVO_2_, venous oxygen saturation; NYHA, New York Heart Association; LVEF, left ventricular ejection fraction

Interestingly, there was a significant association between five-year mortality and FAC measured at the index echocardiogram (OR, 0.91; 95% CI, 0.85–0.97; P = 0.008), but not with TAPSE (OR, 2.79; 95% CI, 0.20–38.0; *P* = 0.44). Nevertheless, no association was found between either FAC or TAPSE and hospitalizations.

## Discussion

In this study of unselected patients with PH, 62% of whom had WHO group 2 disease, conducted at a large tertiary academic center, we found that RVGLS was associated with adverse outcomes, including mortality and hospitalization, after 5 years of follow-up. Compared with the TAPSE and FAC, the RVGLS was also correlated with invasive hemodynamics and was a more robust marker of RV assessment. Although RVGLS predicted outcomes in the overall PH cohort, it did not achieve statistical significance within individual PH subgroups. This may be explained by the smaller sample sizes within each subtype, which likely limited the statistical power to detect significant associations in subgroup analyses.

Prior studies have demonstrated that elevated pulmonary artery pressure is not the primary driver of prognosis in PH patients [18]. The prognosis of PH is driven principally by the adaptation of the RV to the increase in afterload. In this sense, RV assessment is critical for the risk stratification and phenotyping of PH patients [19]. Multiple studies have evaluated different noninvasive methods for the assessment of RV function, including echocardiography, and compared them to the gold standard test, cardiac magnetic resonance imaging [20]. However, the complex RV geometry makes the assessment of RV dimensions and function with two-dimensional imaging challenging. The RV has muscle fibers that predominantly run longitudinally. Thus, most of the contractility in the RV occurs in the longitudinal plane, with the base of the RV moving toward the apex in systole. However, in 2002, Sakuma et al. [21] demonstrated the significant contribution of the percent shortening of the septum–free wall dimension to the RV ejection fraction using cineangiography. The TAPSE is an echocardiographic metric that has been extensively studied in the literature [20, 22–24]. TAPSE is frequently used to assess RV function in patients with PH and has significant prognostic value. However, recent studies suggest that the RVGLS may provide additional information beyond the TAPSE in assessing right ventricular function and prognosis in patients with PH [25].

Both RVGLS and TAPSE are measures of RV function, but they provide different types of information. The TAPSE measures the longitudinal excursion of the tricuspid annulus during systole and is a simple and widely used measure of RV function. However, it measures RV function in one dimension only, which is perhaps not an accurate way of assessing global RV function. It has been shown to be a useful predictor of outcomes in patients with PH and is recommended in guidelines for the evaluation of RV function [26]. RVGLS, on the other hand, measures the deformation of the RV over the cardiac cycle and has been shown to be a more sensitive marker of right ventricular function based on multiple studies [27–29]. Therefore, the lack of correlation between RVGLS and TAPSE in our study, despite a significant association between RVGLS and FAC, likely reflects the differences in what these metrics capture. RVGLS and FAC are both comprehensive measures of global RV contractility, while TAPSE is a one-dimensional measure focused solely on basal longitudinal motion. TAPSE may be less sensitive to regional or global RV dysfunction, especially in cases of septal shift or wall motion abnormalities.

In 2012, a retrospective study by Haeck et al. [12] assessed the prognostic significance of RV longitudinal peak systolic strain (LPSS) in 150 patients with PH compared with FAC and TAPSE. They reported that patients with RV LPSS ≥  − 19% had worse long-term survival (survival rates of 77% at 1 year, 66% at 3 years, and 55% at 5 years). Additionally, they reported that FAC and TAPSE, when used as continuous variables, were not significant predictors of mortality. However, the RV LPSS is a significant determinant of mortality as a continuous and dichotomous variable. Fine et al. [30] evaluated RVGLS in 575 patients with known or suspected PH. They also reported that worsening RV strain quartile was associated with an increased risk of all-cause mortality, PH-related hospitalization, and the need for modified pulmonary vasodilator therapy. In 2010, another study by the Mayo Clinic group assessed RVGLS in 80 patients with newly diagnosed PAH. They reported that strain predicts future RV failure, clinical decompensation, and mortality in patients with PAH [9].

The most extensive data concerning RVGLS are from a meta-analysis published in 2018, which included 10 publications and 1,001 patients with different PH etiologies but predominantly WHO group 1 PAH.

This meta-analysis demonstrated that RVGLS is superior to TAPSE in predicting mortality and poor outcomes. In addition, the findings confirmed the significant prognostic value of the RVGLS compared with the TAPSE when it is treated as a dichotomous and continuous measure of RV function. Interestingly, these findings were consistent across all studies that calculated the strain with or without the interventricular septum [24]. The results of our study are congruent with those of studies demonstrating the incremental utility of RVGLS in RV assessment. We found that mortality was greater at 5 years with worsening RVGLS quartiles and hospitalizations but not with TAPSE. The average TAPSE value in our study was 17 mm, which could explain the lack of association with mortality since a value of less than 15 mm is associated with mortality based on strong evidence [31].

In addition to its utility in predicting poor outcomes in the PH population, RVGLS was found to be correlated with invasive hemodynamics, which could be why it is argued to be a better metric for monitoring RV function in patients with PH. In 2015, Park et al. [10] evaluated the relationships among RVGLS, hemodynamics, and functional assessment in 34 patients with PAH. They reported a significant correlation between RVGLS and cardiac index as well as a correlation between RVGLS and PVR. Furthermore, RVGLS significantly improved after the initiation of therapy, which correlated with improvements in mPAP and PVR in addition to improvements in the NYHA functional class, 6-min walk test, and B-type natriuretic peptide level. In 2015, Vitarelli et al. [13] reported that RV strain using speckle tracking is better than that via conventional echocardiographic metrics, including the TAPSE, for detecting hemodynamic signs of RV failure (a composite of a cardiac index < 2 L/min/m^2^ and an RA pressure > 15 mmHg) in patients with chronic PH and is a better predictor of mortality [13].

In our study, we demonstrated that RVGLS is correlated with invasive hemodynamic numbers. Correlation with invasive hemodynamics is of relevant value in disease monitoring and detection of early RV changes to modify therapy, if necessary. Prior studies have shown that the TAPSE is negatively correlated with the sPAP and PVR and positively correlated with CO. However, accurately obtaining the TAPSE is sometimes challenging in the setting of regional RV wall abnormalities and/or severe tricuspid regurgitation, which does not occur with RVGLS [32].

More importantly, the RV strain is a useful tool for monitoring patients with PAH after therapy initiation and has been shown to be correlated with prognosis and future outcomes [33, 34].Hardegree et al. [35] reported that persistent severe reduction in strain (≤ 12.5%) or worsening strain to that value at 6 months was associated with NYHA functional class III or IV, increased diuretic use, increased mPAP, and decreased survival. In addition, an improvement in RV strain by an absolute value of 5% on therapy was found to be a predictor of better survival, which makes it highly valuable for the serial quantitative assessment of RV function [35]. Prior studies have also demonstrated that the TAPSE can be used to monitor RV function after modifying PAH therapy. One study revealed that a change in the TAPSE from ≤ 15 to > 15 mm after treatment modification was associated with better survival. However, relative changes in TAPSE were not associated with outcome [36].

### Limitations

This was a retrospective analysis of patients with different etiologies of PH. However, WHO groups 1 and 2 are the predominant etiologies. Nevertheless, most of our patients with WHO group 1 disease had other comorbidities (cardiac and/or pulmonary), which made the subgroup analysis of this group of patients not representative of WHO group 1 PAH. As with any retrospective study, we have some missing data that could affect our results, especially some of the measured echocardiographic metric values. Furthermore, three-dimensional speckle-tracking echocardiography a more precise method for measuring RVGLS, was not accessible for all participants in our study. Consequently, RVGLS measurements were not conducted using three-dimensional speckle-tracking echocardiography. A further limitation of the mortality analysis is that time-to-event information and censoring could not be fully addressed, as a time-to-event analysis was not feasible in this cohort due to the unavailability of a consistent index date and follow-up data. Also, hospitalizations were modeled as a continuous outcome using linear regression coefficients, as the number of hospitalizations was treated as a continuous variable. However, alternative approaches, such as negative binomial regression, may have been more appropriate for count data, and this represents a potential limitation. Finally, our study did not include follow-up assessments after adjusting for pulmonary vasodilator therapies. The incorporation of such assessments would significantly increase the value and comprehensiveness of future research.

## Conclusions

RVGLS is an echocardiographic marker that correlates closely with FAC and invasive pulmonary hemodynamics. In this study, both RVGLS and FAC were associated with 5-year mortality, whereas TAPSE was not. Notably, only RVGLS showed a significant association with hospitalization, suggesting that it may provide additional prognostic value in patients with PH.

## Supplementary Information

Supplementary Material 1. Table S1. Correlation between RVGLS and both stroke volume and LVEF, stratified by capillary type (combined, precapillary, and postcapillary pulmonary hypertension)

## Data Availability

Data are available upon request from the corresponding author.
