# Supplementary material for: Predicting outcomes in patients with pulmonary hypertension using right ventricular global longitudinal strain versus tricuspid annular plane systolic excursion (TAPSE) and fractional area change: a retrospective analysis
Source: J Cardiovasc Imaging. 2025 Oct 30;33:15. doi: 10.1186/s44348-025-00059-0 (PMC12573895; doi:10.1186/s44348-025-00059-0)
Supplement: Supplementary file 1 — Supplementary Material 1. Table S1. Correlation between RVGLS and both stroke volume and LVEF, stratified by capillary type (combined, precapillary, and postcapillary pulmonary hypertension) [file 44348_2025_59_MOESM1_ESM.docx]

**Supplementary Table 1.** Correlation between RVGLS and both stroke volume and LVEF, stratified by capillary type (combined, precapillary, and postcapillary pulmonary hypertension)

| Capillary type | Variables | Correlation | P-value |
| --- | --- | --- | --- |
| Combined PH | RVGLS and stroke volume | −0.41 (Pearson) | < 0.001 |
|  | RVGLS and LVEF | −0.33 (Spearman) | < 0.001 |
| Precapillary PH | RVGLS and stroke volume | −0.01 (Pearson) | 0.91 |
|  | RVGLS and LVEF | 0.06 (Spearman) | 0.57 |
| Postcapillary PH | RVGLS and stroke volume | −0.23 (Pearson) | 0.12 |
|  | RVGLS and LVEF | −0.07 (Spearman) | 0.63 |

Abbreviations: RVGLS: right ventricular global longitudinal strain; LVEF: left ventricular ejection fraction; PH: pulmonary hypertension.
